# Taste Changes in a Rat Model of Spinal Cord Injury: Impact of High-Fat Diet and Weight Loss Surgery

**DOI:** 10.3390/nu18030503

**Published:** 2026-02-02

**Authors:** Jonathan Snyder, Tiffany Tang, Gregory M. Holmes, Andras Hajnal

**Affiliations:** Department of Neuroscience and Experimental Therapeutics, College of Medicine, Penn State University, Hershey, PA 17033, USAgmh16@psu.edu (G.M.H.); ahajnal@psu.edu (A.H.)

**Keywords:** spinal cord injury, dietary obesity, weight loss surgery, sleeve gastrectomy, taste preferences, metabolic disorder, dorsal vagal complex

## Abstract

Background: Approximately two-thirds of individuals with spinal cord injury (SCI) become overweight or obese. Weight loss surgery, including vertical sleeve gastrectomy (VSG), is one of the most effective long-term treatments for obesity and type 2 diabetes. Introduction: The main objective of this study was to test in our diet induced obesity rat model whether subjects respond to VSG in the same way as subjects with or without SCI. Methods: To address this question, male Wistar rats underwent either T3 contusion injuries or sham spinal surgeries (Sham). Following recovery, all rats were fed a high-energy, high-fat diet (HFD) for six weeks before undergoing VSG. Taste responsivity and preferences were assessed at multiple time points. Results: Prior to HFD exposure, SCI rats exhibited significantly reduced lick responses for sucrose at higher concentrations and increased licking for low concentrations of sodium, although 2BC sucrose preference was unchanged. HFD feeding in SCI rats enhanced salt and sucrose licking overall. Importantly, VSG reduced sucrose licking, with SCI rats showing greater sensitivity to this effect. cFos immunohistochemistry further revealed enhanced neuronal activation to sucrose ingestion in the dorsal vagal complex, including the rostral subnucleus of the nucleus of the solitary tract. Discussion and Conclusions: Together, these findings support the hypothesis that SCI alters taste functions, thereby increasing vulnerability to diet-induced obesity and that VSG may restore sweet taste responsivity even more effectively in SCI-associated obesity than in non-SCI obesity. Future studies are needed to clarify the neural and hormonal mechanisms mediating these effects and to determine their translational relevance to human SCI populations.

## 1. Introduction

Spinal cord injury (SCI) occurs at a rate of approximately 54 cases per million annually in the United States, imposing substantial costs in terms of life expectancy, healthcare utilization, and multiple determinants of quality of life, with outcomes varying according to injury level and degree of motor function loss [1]. The devastating consequences of SCI are well recognized by both clinical and pre-clinical researchers: derangements in somatic and autonomic neural control are compounded by reduced physical activity, ultimately leading to dysregulation of energy homeostasis that rapidly progresses to obesity (reviewed in [2]). Indeed, the likelihood of developing obesity is estimated to be 16–44% higher in individuals with SCI compared with age-matched counterparts without SCI [3,4,5].

Extreme obesity is typically defined by a body mass index (BMI) exceeding 40 kg/m^2^. However, the validity of BMI in SCI populations is debated, as it may underestimate adiposity following injury (see [2] for detailed discussion). Regardless, due to a disproportionately elevated fat-to-lean mass ratio, individuals with SCI exhibit a range of obesity-associated complications, including glucose dysregulation [6,7], insulin resistance, and cardiovascular disease. Collectively, these comorbidities, often described as metabolic syndrome [8], occur at higher rates in the SCI population than in the general population.

Obesity remains notoriously difficult to treat due to its multifactorial origins and the complex physiological systems involved. Until the recent introduction of glucagon-like peptide 1 receptor agonists (GLP-1RAs), bariatric surgery—also termed metabolic or weight loss surgery (WLS)—was the only consistently effective intervention for obesity and related metabolic disease. The two most common procedures, Roux-en-Y gastric bypass (RYGB) and vertical sleeve gastrectomy (VSG), have accounted for more than 200,000 operations annually in the U.S. over the past two decades [9]. More recently, VSG has surpassed RYGB due to its relative simplicity, improved tolerability, and cost-effectiveness. Clinical evidence for obesity treatment in individuals with SCI, however, remains sparse beyond dietary and exercise-based interventions. While GLP-1RAs may hold promise due to their beneficial effects on glycemic control, inflammation, and cardiometabolic health [10], their widespread use for weight loss—as seen in the general population—could pose specific risks in SCI. These include gastrointestinal dysfunction (e.g., gastroparesis, impaired motility [11,12]) and pancreatic abnormalities affecting both endocrine and exocrine functions [13,14]. Although limited observational data suggest potential benefits [15], the efficacy and safety of WLS in individuals with SCI remain understudied.

The precise mechanisms underlying the effects of WLS are incompletely understood. Neither mechanical restriction, malabsorption, nor altered hormonal signaling alone fully explain the surgery’s capacity to reduce food intake and improve eating behaviors [16,17,18]. Growing evidence indicates that shifts in food and taste preferences, as well as improvements in gut dysbiosis, play an important role [18,19,20,21,22,23,24]. Following WLS, many patients spontaneously decrease their intake of calorie-dense, highly palatable foods [25,26,27,28]. Similar patterns are observed in rodents, with post-surgical reductions in preference for high-fat and high-sugar foods, leading to a normalization of food choices and eating behaviors comparable to lean controls [20,27,29,30,31,32]. While these phenomena are of clear clinical significance, their occurrence has not yet been systematically evaluated in individuals with SCI or in pre-clinical SCI models.

To address this gap, we propose that VSG may provide effective weight loss while also conferring advantageous changes in food preference in the context of SCI. In a recent feasibility study [33], we demonstrated that obese, high-fat diet (HFD)-fed rats subjected to SCI tolerate VSG well and experience superior weight loss outcomes compared to non-SCI controls [33]. Extending this work led to the hypothesis for the present study that in this model of obese SCI rats, altered taste functions may be present, and the benefits from VSG—at least partially—are related to improvements in primary taste functions and preferences.

## 2. Materials and Methods

**Figure 1:**
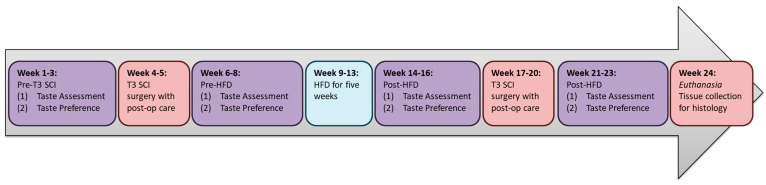
Experimental timeline.

Animals and timeline: adult male Wistar rats (n = 22, 200–240 g upon receipt; Envigo, Indianapolis, IN, USA) were initially housed in pairs or triads. The experimental timeline is delineated in Figure 1. Briefly, after acclimatizing to the facility for one week following shipment, rats were tested for baseline taste responsivity for sucrose (Fisher Scientific, Fair Lawn, NJ, USA) and sodium chloride (NaCl; Fisher Scientific, Fair Lawn, NJ, USA). Each taste testing session requires approximately two weeks. Following the T3 SCI surgery or sham laminectomy, the rats were housed individually. After recovering from SCI or sham laminectomy surgery for 2 weeks, rats were retested for taste responsivity. Animals were then switched from a standard chow diet (Teklad 2018, 6.2% kcal from fat, 4.00 kcal/g; Envigo) to a high-fat diet (HFD; 60% kcal from fat, 5.13 kcal/g; Research Diets, New Brunswick, NJ, USA). After 5 weeks of HFD as their only source of caloric intake, taste responsivity was reassessed. Following this reassessment, animals were given either a sham abdominal surgery or VSG and retested for taste functions after three weeks of recovery. Animals were randomly selected for either surgery such that within each SCI injury state, seven animals received VSG. This number was arrived at based on previous power analysis and attrition rates from SCI-injured animals receiving VSG and their subsequent weight loss [33]. Animals were removed from the study if their body weight fell below 20% of their weight prior to HFD exposure. During all behavioral experiments, the experimenter was blinded to the abdominal surgery state while blinding to the SCI was not feasible. For all treatments and taste or ingestive stimuli, the same order with equal access across groups was conserved to minimize confounding. Unless otherwise noted, food and water were provided ad libitum, and animals were maintained on a 12 h light/dark cycle. All procedures were in accordance with the National Institutes of Health Animal Welfare Guide and were approved by the Institutional Animal Care and Use Committee of the Penn State University College of Medicine (protocol title: Mechanisms of gastrointestinal neuropeptide signaling in an animal model of spinal cord injury following weight loss surgery; Protocol #: PRAMS201747686, first approval date: 2017, last approval date: 17 June 2025, approved for 3 years) and related current US Army Animal Care and Use Review Office (ACURO) protocol (#SC230094.e00) in accordance with DOD Instruction 3216.01, “Use of Animals in DOD Conducted and Supported Research and Training”, and US Army Regulation 40–33, “The Care and Use of Laboratory Animals in DOD Programs”.

Brief-access lick assessment: taste assessments were made using the Davis Rig automated lickometers from Med Associates as used broadly and previously published in our lab [34,35]. This automated system presents different taste solutions in a randomized balanced sequence and detects licks through insensible electrical conduction. Using an automated door, the system controls the rats’ access to each taste solution, limiting it to 10 s after the first detected lick (a trial) such that orosensory taste is the primary driver of licking behavior rather than satiation or other post-ingestive feedback. Concentration curves were constructed using common ranges of sucrose (sweet) and NaCl (salty). Brief-access licking data was compiled and analyzed using Python (Version 3.13) scripts to detect licking bouts as defined by a minimum of three licks with no more than 350 ms between any licks and ceasing upon any interval greater than 350 ms. This analysis generates metrics such as average bout length, non-bout licks, and in-bout lick frequencies. Animals were excluded from analysis if they failed to initiate at least three trials in total as an indication of failure to learn the paradigm (n = 2).

Two-bottle choice test: to test ingestive preference in a long access setting to be inclusive of post-ingestive effects, the two-bottle choice test was used over a period of 24 h with 0.3 M sucrose.

T3 spinal cord injury model: all surgical procedures were performed under isoflurane anesthesia (3–5% in 1 L/min O_2_) to achieve areflexia as previously published [33]. Briefly, a prophylactic subcutaneous dose of enrofloxacin (10 mg/mL; Bayer, Leverkusen, Germany) was given to reduce post-surgical infection risk, and animals received a single pre-operative dose of sustained-release buprenorphine (1 mg/kg; Reckitt Benckiser Pharmaceuticals, Richmond, VA) for analgesia. Following T3 spinal exposure, a 300-kilodyne contusion injury (15 s dwell time) was delivered to the T3 spinal level. Surgical controls underwent identical laminectomy and spinal exposure without contusion. Postoperative care included daily monitoring of body weight, food intake, bladder expression, and wound status, as well as provision of fluids and inspection for complications related to reduced mobility and weight gain.

Vertical sleeve gastrectomy: this procedure was identical to our previously published protocol [33]. Briefly, in isoflurane-anesthetized rats, a midline laparotomy was performed to expose the ventral stomach. The stomach was then divided using a 35 mm straight endocutter (ENDOPATH ETS; Ethicon Endo-Surgery, Inc., Cincinnati, OH, USA). The staple line was placed 2–3 mm below the gastroesophageal junction on the lesser curvature and extended along the greater curvature to create a tubular gastric pouch (~20% of the original stomach volume). The abdominal incision was closed, and rats received subcutaneous saline (50 mL/kg). Following vertical sleeve gastrectomy (VSG), rats were provided a clear liquid diet (Boost^TM^; Nestlé, Anderson, IN, USA, ad libitum) for one week to promote gastric wound healing and then returned to their assigned diet for 6 additional weeks. Postoperative care included daily monitoring of body weight and diet intake, wound care as needed, and inspection for complications (e.g., cellulitis, edema).

cFos immunohistochemistry for visualizing neural activity following sucrose consumption: at the end of the study, rats were trained on four consecutive days to drink 5 mL of 0.9 M sucrose within 30 min. After this exposure, rats that successfully drank the full amount on the final day were anesthetized and perfused with saline and then paraformaldehyde (one rat was excluded for failure to drink the full volume of sucrose). For c-Fos IHC, brains were sectioned in 40-micron sections, and sections were washed with PBS three times for 5 min on shaker at room temperature. Tissue sections were blocked with 5% goat-serum in 0.4% Triton-X/PBS for one hour at room temperature and then rinsed for five minutes three times with PBS. Following blocking, tissue sections were incubated for 24 h overnight at room temperature in cFos primary antibody (c-Fos rabbit polyclonal IgG; Santa Cruz, SC-52, Dallas, TX, USA) at a dilution of 1:10,000 using 0.4% Triton-X/PBS and goat serum. After primary antibody incubation, sections were washed and incubated for two hours at room temperature in biotin-goat anti-rabbit IgG secondary antibody (Santa Cruz, SC-2040) at a 1:200 dilution with 4% Triton-X/PBS and goat serum. After secondary antibody incubation, sections were washed three times for five minutes in PBS. C-fos immunoreactivity was visualized using an immunocytochemical avidin–biotin complex (ABC) staining technique; ABC peroxidase (VECTASTAIN Elite ABC HRP Kit, Vector Laboratories^®^, Burlingame, CA, USA) was prepared at 1:1000 for 30 min before tissue sections were incubated in it for one hour at room temperature. Tissue sections were then washed three times for five minutes each in PBS and then incubated in a diaminobenzidine (DAB)/nickel ammonium sulfate solution for five minutes until sections turned a light to medium purple. Sections were then washed in PBS three times for five minutes each wash and then transferred to phosphate buffer. Sections were then mounted onto gelatin-coated slides, dried overnight, and put through a series of dehydration steps with 100% ethanol and xylene. Slides were then cover-slipped with Permount^®^ Mounting Medium (Fisher Chemical^TM^; cat# SP15–100, Fair Lawn, NJ, USA) and left to dry overnight.

Microscopy and cFos immunohistochemistry: an Olympus^®^ BX50 light microscope (Waltham, MA, USA) with Olympus^®^ CellSens Software (Version 1.16) was used to capture 20× images of the caudal (−14.30 to −14.08 mm from the bregma), intermediate (−13.68 to −13.80 mm from the bregma), and rostral (−12.30 to 13.30 mm from the bregma) dorsal vagal complex (DVC) for data analysis [36]. For each rat, two sections of each DVC subregion were imaged—for every individual section, a right-side and a left-side image was obtained for cFos-positive cell counting analysis.

Image analysis: ImageJ Software (Rasband, W.S., ImageJ, U. S. National Institutes of Health, Bethesda, MD, USA; Version 1.53) and *The Rat Brain in Stereotaxic Coordinates: 4th Edition* [36] were used to create standard regions of interest (ROIs) for tissue section images. Once each image was assigned an accurate ROI, c-Fos-positive cells were manually counted using the ImageJ multi-point tool. After cFos positive cells were counted on each image, the ImageJ analyze tool was used to measure the total amount of cells counted. Cells were counted by two different individuals. Statistical analysis and graph generation used an average count of cFos-positive cells for each subregion counted on both the left and right sides.

Statistical analysis: statistical analysis and graphing was performed primarily in GraphPad Prism version 10.5 (GraphPad Software, San Diego, CA, USA). Assumptions of normality were tested by Shapiro–Wilk and Kolmogorov–Smirnov tests. Licking data for groups differentiated by SCI and diet were analyzed by mixed effects ANOVA where the factors were SCI (between), HFD (within), and tastant (within). Later, when groups were differentiated by both SCI and VSG status, a three-way mixed-effects ANOVA was used to identify main effects and interactions where the effects were SCI (between), VSG (between), and tastant (within). Post hoc multiple comparisons were made for the between factors (surgical interventions) with Bonferonni corrections. Simple linear regression was performed for spout discrimination, and those regressions were compared in R for all three factors (tastant concentration * SCI * VSG). For cFos data, individual 2-way ANOVA was performed within each subnuclei of the NTS. All data in this paper is presented as mean ± S.E.M. Partial η^2^ values are included for all statistically significant main effects, and Cohen’s D values are included for all statistically significant t-tests. Partial η^2^ values less than 0.05 were considered small, 0.05 to 0.12 were considered medium, and above 0.12 was considered large.

## 3. Results

### 3.1. Taste Responsivity Following SCI

**Figure 2:**
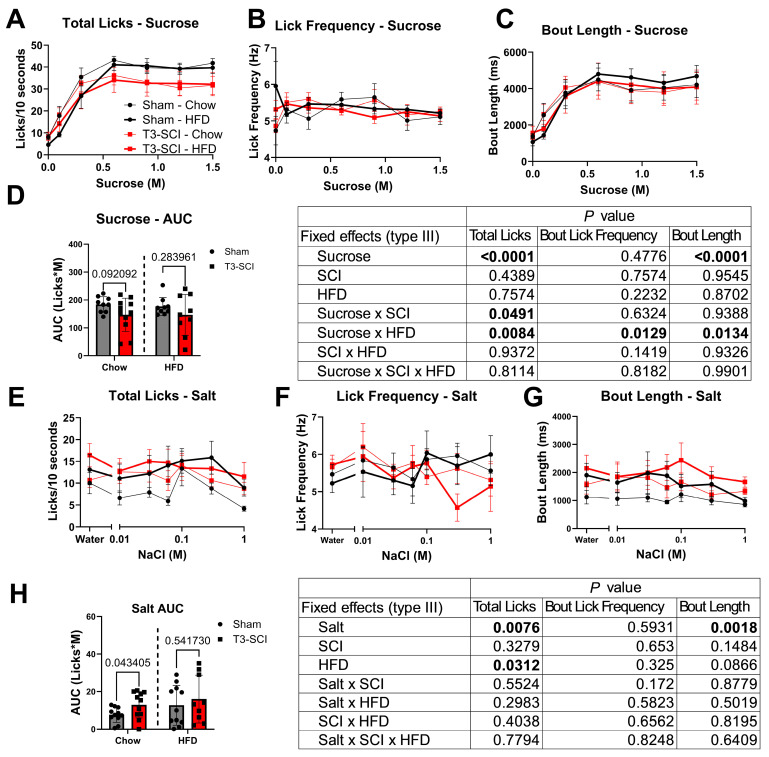
SCI reduces responsivity to sucrose but enhances responsivity to salt. (**A**) Sucrose trials: T3-SCI rats showed fewer total licks at higher sucrose concentrations (**B**) Lick frequency in response to sucrose shows SCI and HFD do not modify oromotor coordination. (**C**) HFD modified bout length and sucrose motivation. (**D**) AUC of lick responses demonstrates a negative trend from SCI. (**E**) Rats displayed greater total licks after HFD exposure across NaCl concentrations, consistent with enhanced salt response despite (**F**) unchanged lick frequency. (**G**) Bout length varied with salt concentration. (**H**) Area under the curve for total licks shows increased salt intake during short access in T3-SCI rats prior to HFD. Error bars represent ± SEM. Statistics were performed at the trial level. For more details, refer to the text.

Behavioral taste testing under normal chow and HFD conditions revealed that SCI modestly reduced sucrose licks while enhancing responsiveness to salt. In sucrose trials (Figure 2A–D, n = 9–11 rats), both groups increased licking with rising sucrose concentrations (main effect of sucrose: F(6, 114) = 74.61, *p* < 0.0001, partial η^2^ = 0.7972), but T3-SCI rats performed significantly fewer total licks than sham controls in response to increasing sucrose concentration (sucrose × SCI: F(6, 86) = 2.689, *p* < 0.05, partial η^2^ = 0.1579). The deficit was most pronounced at 0.9 M, 1.2 M, and 1.5 M sucrose (Figure 2A), indicating attenuated sweet motivation. Lick frequency (Figure 2B; n = 9–11 rats) demonstrated an interaction between HFD and sucrose concentration (sucrose × HFD: F(6, 92) = 2.879, *p* < 0.05, partial η^2^ = 0.1581) but was not modified by SCI. Bout-length analysis (Figure 2C; n = 9–11 rats) revealed longer licking episodes at moderate sucrose concentrations as a consequence of HFD (sucrose × HFD: F(6, 85) = 2.876, *p* < 0.05, partial η^2^ = 0.1683), implying greater persistence once consumption was initiated after the HFD exposure period. AUC analysis is displayed for total lick response normalized to concentration to display trends in overall response to oral sucrose stimulation (Figure 2D). In contrast, salt trials (Figure 2E–H) demonstrated an opposite trend. Total licks varied with NaCl concentration (main effect of salt: F(6, 120) = 3.084, *p* < 0.01, partial η^2^ = 0.1336; n = 9–11 rats) and were markedly higher following HFD exposure (HFD: F(1, 20) = 5.370, *p* < 0.05, partial η^2^ = 0.2117), reflecting enhanced salt responsivity. Lick frequency (Figure 2F; n = 9–11 rats) remained unchanged, consistent with preserved oromotor coordination. Bout lengths also remained unchanged despite modest trends for SCI and HFD. AUC analysis for total lick response normalized to salt concentration reveals SCI animals exhibited an increased exposure limited to the chow diet period (Figure 2H, *p* < 0.05, Cohen’s D = 0.7569). Overall, these results indicate that SCI changes taste responsivity with medium to large effect size with regard to sucrose taste response, which is further modified by HFD approximately equally across SCI groups. The medium-to-large effect sizes of sucrose × SCI and sucrose × HFD without significant post hoc differences at specific concentrations suggest a global shift in appetitive drive without a specific concentration threshold effect. SCI diffusely increases motivation for salty stimuli (Figure 2H), suggesting a shift in gustatory balance toward sodium appetite and altered brainstem processing of taste-reward signals after injury.

### 3.2. Body Weight Following VSG

**Figure 3:**
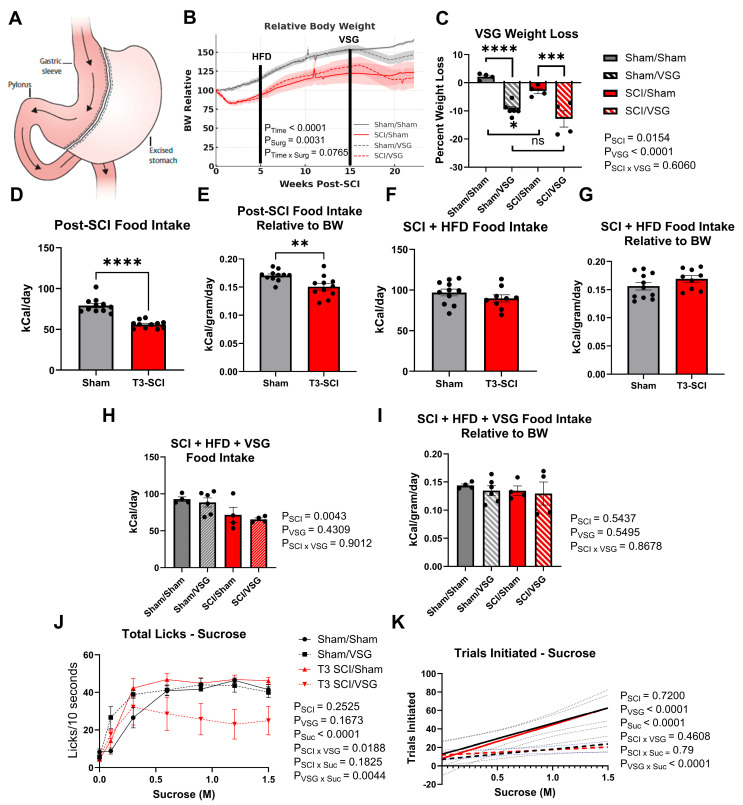
VSG induces weight loss and reduces sucrose responsivity. (**A**) Schematic illustration of the VSG procedure showing excision of approximately 80% of the stomach along the greater curvature to create a narrow gastric sleeve. Arrows indicate the directionality of the GI tract. (**B**) Post-SCI body weight trends, shown as percent baseline, throughout the entire study period. Both SCI groups (SCI/Sham, SCI/VSG) lost and maintained weight at significantly lower level than the non-SCI. (**C**) Percent weight loss measured five weeks after surgery in spinal-intact (Sham/Sham, Sham/VSG) and T3 spinal cord-injured (SCI/Sham, SCI/VSG) rats. Both VSG groups showed significant weight reduction relative to their sham-operated counterparts, confirming surgical efficacy. (**D**,**E**) Total caloric intake and total caloric intake relative to body weight from normal chow following the T3-SCI injury averaged across the test days for gustometry data. (**F**,**G**) Total caloric intake and total caloric intake relative to body weight from HFD chow following averaged across the test days for gustometry data. (**H**,**I**) Total caloric intake and total caloric intake relative to body weight from HFD chow following recovery from VSG averaged across the test days for gustometry data. (**J**) Total licks during brief-access testing across sucrose concentrations (0.1–1.5 M). VSG rats displayed markedly fewer licks at higher sucrose concentrations (≥0.6 M) compared with sham controls, indicating reduced hedonic and consummatory drive. (**K**) Linear regression of trial initiation as a function of sucrose concentration. The shallower slopes observed in VSG groups reflect reduced motivation to engage with sweet stimuli. Symbols: * *p* < 0.05, ** *p* < 0.01, *** *p* < 0.001, **** *p* < 0.0001.

As illustrated in Figure 3A, the VSG procedure removed approximately 80% of the stomach along the greater curvature, producing a tubular gastric sleeve that restricts volume and alters nutrient signaling. As expected, therefore, five weeks post-VSG, both spinal-intact (Sham/VSG) and T3 SCI/VSG rats displayed substantial relative reductions (a large effect size) in body weight compared with their respective sham controls (main effect of VSG: F(1,14) = 49.09, *p* < 0.0001, partial η^2^ = 0.778; Figure 3C). Although SCI animals overall weighed less, maintaining a 10–20% overall weight loss since the time of surgery (Figure 3B) than their spinal-intact counterparts, there was no significant interaction between VSG and SCI (F(1, 14) = 0.28, *p* > 0.05), indicating that the magnitude of postoperative weight loss was similar in both neurological conditions. Thus, VSG induced a reliable and sustained reduction in body mass regardless of spinal injury status, confirming the procedure’s metabolic efficacy in this model and establishing a robust physiological framework for interpreting subsequent behavioral and neural outcomes. Food intake during the three periods of taste study is summarized within each taste testing period. SCI strongly suppresses both absolute caloric intake (Figure 3D, Cohen’s D = 3.20) and relative caloric intake (Figure 3E, Cohen’s D = 1.32). HFD and time appear to normalize this effect on caloric intake (Figure 3F,G), and no differences were detected during the gustometry period following VSG (Figure 3H,I).

### 3.3. Taste Responsivity Following VSG

Taste-driven licking behavior revealed that VSG markedly diminished responsivity to high-concentration sucrose. Across concentrations from 0.1 M to 1.5 M, total licks (Figure 3J) increased with sucrose concentration in all groups (main effect of sucrose: F(6, 33.01) = 58.56, *p* < 0.0001, partial η^2^ = 0.8185) but were significantly suppressed by VSG (sucrose × VSG F(2.539, 33.01) = 5.805, *p* = 0.0044, partial η^2^ = 0.3049). Moreover, a robust SCI × VSG interaction (F(1, 13) = 7.191, *p* < 0.05, partial η^2^ = 0.3561) indicated an additive dampening of sweet-driven licking in the T3 SCI/VSG group. As with previous taste response data, no specific concentration was significantly different, indicating a strong global response across the concentration curve without focal concentration sensitivity shifts.

Complementary regression analysis of trial initiation across sucrose concentrations (Figure 3K) demonstrated that VSG animals initiated significantly fewer trials as sucrose concentration increased (main effect of VSG: F(1, 88) = 84.05, *p* < 0.0001), resulting in a shallower behavioral slope compared with sham controls, while SCI alone exerted no effect. Together, these findings indicate that VSG reduces both consummatory and appetitive components of sucrose reward, pointing to a surgery-induced attenuation of orosensory–motivational drive that persists in the presence of spinal cord injury.

### 3.4. Long-Access Sucrose Preference

**Figure 4:**
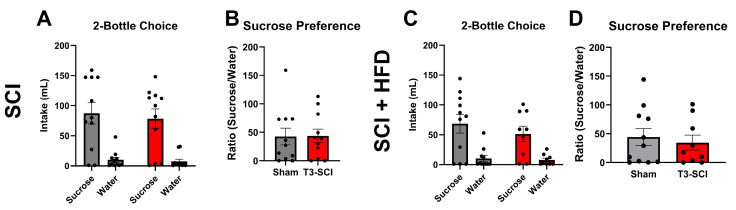
Long-access sucrose preference following SCI and HFD. (**A**,**B**) Under chow conditions, SCI and sham rats exhibited comparable 24 h sucrose and water intake and similar sucrose preference ratios calculated as sucrose intake over 24 h divided by water intake during the same period. (**C**,**D**) HFD increased total intake across groups but did not change sucrose preference between SCI and sham rats. Bars represent mean ± SEM.

Long-access two-bottle choice testing across experimental conditions revealed no significant effects of SCI or interaction between SCI and HFD on 24 h sucrose consumption and preference. Under normal chow conditions, there were no significant differences between sham-operated and SCI rats in total 0.3 M sucrose intake (t(18) = 0.3701, *p* > 0.05) or water intake (t(18) = 0.4431, *p* > 0.05; Figure 4A). Likewise, the ratio of sucrose-to-water intake (sucrose preference) was comparable between groups (t(8) = 0.5993, *p* > 0.05; Figure 4B). Following six weeks of HFD exposure, both sham and SCI rats maintained similar total sucrose (t(18) = 0.8102, *p* > 0.05) and water intakes (t(18) = 0.4082, *p* > 0.05; Figure 4C), with no significant differences in sucrose preference ratios (t(18) = 0.4746, *p* > 0.05; Figure 4D). Collectively, these findings indicate that neither SCI nor HFD alone alters long-term sucrose preference.

### 3.5. DVC Activation Following Sucrose Consumption

**Figure 5:**
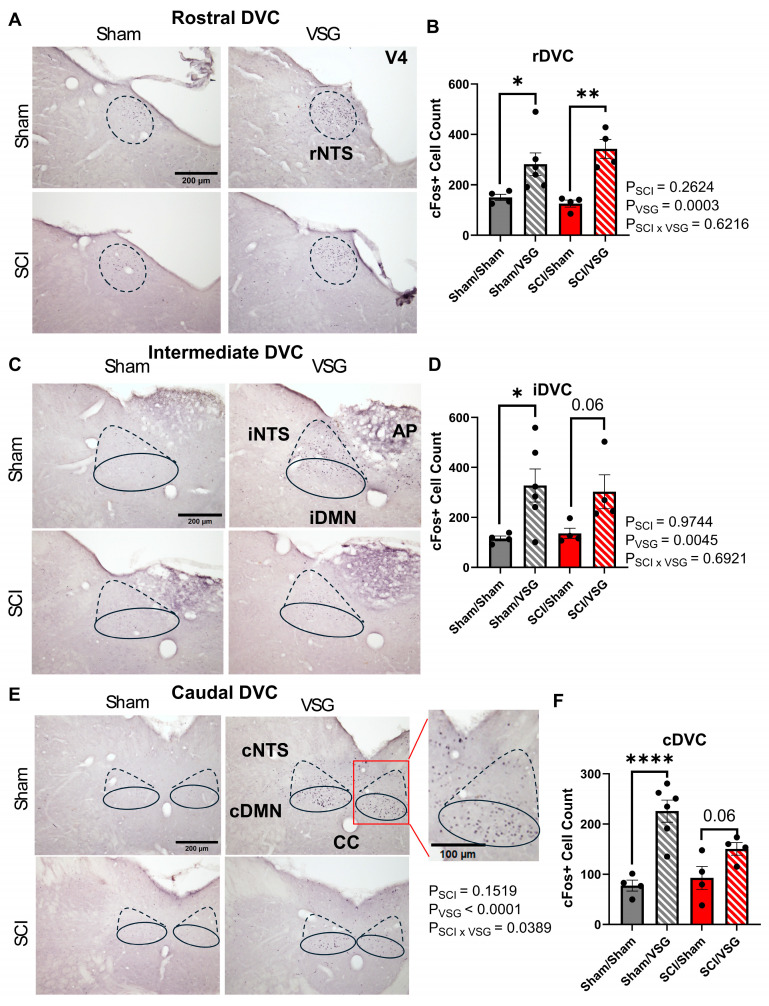
VSG increases neuronal activation across levels of the DVC. (**A**) Representative photomicrographs showing cFos immunoreactivity within the rostral DVC across Sham and SCI groups with or without VSG wherein the NTS is approximately indicated by dashed lines. (**B**) Quantification of cFos^+^ cell counts in the rostral DVC (n = 4–6/group). (**C**) Representative images of cFos labeling in the intermediate DVC wherein the NTS is approximately indicated by dashed lines, and the DMV is indicated by the solid lines; (**D**) quantification of cFos^+^ cells (n = 4–6/group). (**E**) Representative cFos labeling in the caudal DVC wherein the NTS is approximately indicated by dashed lines, and the DMV is indicated by the solid lines; (**F**) corresponding quantification (n = 4–6/group). Bars represent mean ± SEM. 4V: 4th ventricle, CC: central canal. Symbols: * *p* < 0.05, ** *p* < 0.01, **** *p* < 0.0001. Error bars represent ± SEM. For statistical analysis performed at trial level, refer to the text.

Immunohistology was performed to visualize cFos immunoreactive neurons activated by 30 min access to consume a fixed-amount 0.9 M sucrose solution. The area of interest for the analysis was the dorsal vagal complex (DVC), which is inclusive to the nucleus of the tractus solitarius (NTS), the first central relay of afferent taste and visceral projections, the dorsal motor nucleus of the vagus (DMV), the main source of efferent projections to the periphery, and the area postrema (AP) known as a major chemosensory area integrating peripheral and central signals. Representative cFos-immunoreactive sections (Figure 5) show visibly denser labeling within the rostral (rDVC, Figure 5A,B), intermediate (iDVC, Figure 5C,D), and caudal (cDVC, Figure 5E,F) subnuclei in VSG rats compared to sham cohorts, irrespective of SCI status. Quantitative cell counts confirmed significant main effects of the brain region (F(1.51, 21.14) = 9.72, *p* = 0.002, partial η^2^ = 0.7170), and analysis was therefore performed within each region as a two-way ANOVA. In each subregion, VSG rats showed elevated cFos expression (*p* < 0.05–0.001), particularly within the rostral subnucleus (main effect of VSG: F (1, 14) = 22.74, partial η^2^ = 0.6192), primarily consisting of the rostral region of the NTS that receives gustatory afferents (Figure 5A,B). Notably, however, cFos immunoreactivity was also significantly different, although to a lesser extent, in the cDVC which receives primarily visceral afferents (Figure 5E,F: main effect of VSG: F (1, 14) = 27.01, partial η^2^ = 0.6590, SCI × VSG: F (1, 14) = 5.190, partial η^2^ = 0.2705) and also in the intermediate areas of DVC which has mixed afferents and efferents (Figure 5C,D: main effect of VSG: F (1, 14) = 11.39, partial η^2^ = 0.4487). Interestingly, as opposed to the more rostral areas, within the cDVC, the majority of cFos-IR neurons appeared in the DMV instead of the NTS (Figure 5E), pointing to a change occurring in efferent activity of the vago–vagal reflexes. Notably, statistical analysis revealed a significant interaction for SCI × VSG in this area, strongly suggesting that the changes in efferent vagal activity are uniquely related to SCI. Overall, the histological results demonstrate that sleeve gastrectomy enhances both oral and post-ingestive sensory signaling to the DVC while concurrently reducing behavioral responses to sweet tastants. The effect sizes of VSG across DVC subnuclei represent a large effect. Functionally, this dissociation suggests that VSG amplifies early hindbrain satiety and/or aversive feedback mechanisms that suppress responsivity and, in turn, intake of sweet solutions, a process that remains intact following SCI.

## 4. Discussion

This study demonstrates that thoracic SCI alters primary taste-guided behavior in rats and that these alterations are further modulated by an obesogenic diet and by VSG. From the results of the study, three major findings emerge. First, SCI selectively blunts sucrose-driven licking while enhancing salt responsivity, without microstructural changes indicating altered motivational dynamics rather than gross oromotor dysfunction. Second, HFD exposure equally modifies lick responses to both sucrose and salt. Third, VSG produces robust relative weight loss in both SCI and sham rats and selectively suppresses licking for sucrose. This effect was accompanied by heightened sucrose-evoked cFos activation in DVC subnuclei. Together, these findings support a model in which SCI shifts gustatory preferences (reduced sweet, increased salt), HFD increases overall taste-driven lick responses, and VSG then reverts taste responses towards pre-obese levels despite continued HFD feeding by increasing sensitivity of DVC afferent signaling to recalibrate hindbrain processing to suppress motivation for sweet.

### 4.1. SCI Alters Taste Responsivity

Under normal chow, SCI reduced sucrose licking at higher concentrations, while salt licking increased at low–moderate NaCl concentrations. The divergence between brief-access (10 s) and long-access (24 h) sucrose intake, i.e., microstructure changes without a change in total daily preference, implies that SCI primarily modifies short-latency orosensory–motivational processes rather than overall intake. This pattern aligns with known SCI-related dysregulation of autonomic and visceral feedback [13] and emerging evidence for pancreatic/exocrine alterations after SCI [14] which could alter central representation of taste valence and interoceptive state. Such effects could have contributed to our observation of activation of visceral efferent neurons within the DVM in the caudal DVC following SCI. The very large effect sizes observed in the DVC compared to the medium-to-large effect sizes in behavioral taste data point to a significant initial neural signal with a densely integrated and noisy network prior to behavioral outcomes. Enhanced salt preferences may reflect homeostatic adaptations in sodium appetite or altered visceral and somatosensory functions after injury, consistent with evidence implicating DVC (NTS, AP)–parabrachial nucleus (PBN)–hypothalamic circuits in sodium regulation [37,38]. In contrast, reduced sweet preferences likely reflect a diminished hedonic drive or altered predictive coding of caloric value, in line with prior studies showing impaired sweet-reward processing after obesity, as well as peripheral or central neural injury [39,40,41]. This may point to greater translational value of VSG to individuals with SCI who primarily struggle with hedonic eating motivation.

Taste function after SCI remains largely unexplored, but converging indirect evidence suggests that both orosensory processing and its integration with visceral feedback may be disrupted. Clinical studies in individuals with spinal cord injury have reported altered chemosensory perception, including changes in olfactory thresholds and diminished flavor intensity, although rigorous quantitative assessments of gustatory sensitivity and preference are lacking [42]. More broadly, injury to peripheral sensory nerves is known to disrupt taste sensation, leading to hypogeusia, ageusia, or phantom oral perceptions [43]. Experimental studies of localized gustatory nerve lesions—particularly involving the chorda tympani and glossopharyngeal nerves—demonstrate that damage to taste afferents induces measurable deficits in taste responses and reorganization of central gustatory circuits [44,45]. In parallel, extensive evidence indicates that spinal cord injury perturbs visceral and autonomic sensory feedback, including vagal afferent signaling, gastrointestinal motility, and neural processing within the nucleus of the solitary tract, all of which could secondarily modulate gustatory integration [13,14,46,47,48,49].

Relevant to this point, our observation of enhanced sucrose-evoked cFos activation across DVC subregions—including the NTS and motor neurons of the dorsal motor nucleus of the vagus (DMV)—supports a model in which VSG partially restores or amplifies early hindbrain sensory–visceral signaling following SCI. Importantly, the DVC occupies a privileged position at the interface of gustatory input, gastric mechanosensation, and autonomic output. Augmented activation in this circuit is therefore well positioned to influence ingestive behavior by strengthening the coupling between orosensory cues and post-ingestive feedback, thereby constraining intake and recalibrating preference rather than simply suppressing consumption through aversive mechanisms. Consistent with this framework, it has been hypothesized that individuals with SCI may experience impaired satiation due, in part, to a reduced perception of gastric fullness [50]. Our DVC activation data raises the possibility that VSG enhances vagally mediated stretch and nutrient signaling to the hindbrain, improving the fidelity of early satiation signals that are normally degraded after injury.

While our study does not directly assess taste thresholds or hedonic valuation per se, the combination of normalized sucrose intake, altered preference, and enhanced sucrose-evoked DVC activation is consistent with a model in which post-surgical improvements in hindbrain sensory integration promote more appropriate alignment between sensory input, visceral feedback, and feeding decisions. These findings highlight the DVC as a critical substrate through which bariatric surgery may exert beneficial effects on ingestive control after SCI and underscore the need for future studies explicitly quantifying gustatory sensitivity, sensory–reward integration, and vagal afferent function in SCI models.

### 4.2. HFD Amplifies Sweet Taste Responsivity and Masks Some SCI Effects

HFD increased sucrose licking across groups and partially reversed the SCI-associated reduction in sweet licking, as well as the augmented salt preferences. Diet-induced adiposity is known to influence orosensory and reward circuits and to alter gut–brain signaling, microbiota, and endocrine milieu [21,27,51]. Notably, long-access sucrose intake remained similar across groups, again suggesting that moment-to-moment motivational parameters rather than absolute daily consumption were most affected.

### 4.3. VSG Suppresses Sweet-Motivated Behavior While Enhancing DVC Activation

VSG produced substantial weight loss in both SCI and sham animals and selectively reduced sucrose-driven licking, with the steepest suppression at higher concentrations and a marked reduction in trial initiation. These findings are consistent with the literature on humans and rodents showing post-surgical reductions in preference for calorie-dense sweet/fatty foods and normalization of eating patterns [17,19,24,29,30,31]. Notably, the enhanced sucrose-evoked cFos expression observed across DVC subnuclei after VSG appears paradoxical in light of the attenuated behavioral responses. However, rather than reflecting increased hedonic drive, we interpret this dissociation as evidence that VSG restores or amplifies early hindbrain sensory–visceral signaling that is blunted by obesity and further disrupted after SCI, as demonstrated in prior studies of high-fat diet exposure and gastric surgery. Strengthened activation within the DVC, an integrative hub for gustatory input, vagal afferent signaling, and autonomic output, would be expected to increase the gain and salience of anorexigenic hindbrain signals, thereby constraining intake and normalizing preference despite reduced overt consummatory behavior. In this framework, enhanced DVC recruitment reflects improved coupling between orosensory cues and post-ingestive feedback rather than a direct enhancement of palatability. Such early sensory–visceral recalibration provides a parsimonious explanation for the selective suppression of high-concentration sucrose intake and reduced trial initiation, consistent with the idea that bariatric surgery reweights ascending hindbrain signals that shape downstream processing in parabrachial and forebrain reward circuits, rather than acting solely through mechanical restriction [31,51,52,53,54,55]. Potential mediators of this process include altered vagal afferent tone, changes in gastric emptying, and surgery-induced modulation of gut-derived signals (e.g., GLP-1, PYY, CCK, ghrelin), bile acid signaling, and the intestinal microbiome [16,21,56,57], all of which converge on DVC circuitry and are well positioned to influence early satiation and taste–reward integration.

### 4.4. Neuroplasticity After Spinal Cord Injury

Substantial evidence indicates that spinal cord injury triggers widespread neuroplastic reorganization extending beyond the lesion site, affecting both spinal and supraspinal circuits. Filipp et al. [58] comprehensively reviewed molecular and circuit-level plasticity following SCI, emphasizing altered synaptic connectivity, axonal sprouting, and compensatory rewiring within spared pathways. These changes have been documented across sensorimotor, autonomic, and affective domains and can extend into the brainstem and forebrain [59]. Functional imaging and electrophysiological studies also reveal adaptive and maladaptive plasticity in thalamic and cortical sensory networks after SCI [60], suggesting that reorganization of ascending and descending projections may modify the integration of interoceptive and gustatory information. Moreover, SCI induces profound changes in brainstem nuclei receiving vagal and visceral inputs, including the NTS, parabrachial nucleus, and hypothalamus, regions intimately involved in taste and energy balance regulation [61,62]. Thus, although direct evidence for gustatory pathway plasticity post-SCI is lacking, the extensive remodeling observed in related sensory and autonomic systems provides a strong mechanistic basis for hypothesizing altered taste processing in this context.

### 4.5. Clinical and Translational Implications

Clinically, obesity, dysglycemia, and cardiometabolic disease occur at higher rates after SCI [2,7]. While GLP-1 receptor agonists have promise in SCI for neuroimmune/metabolic benefits [10], gastrointestinal dysmotility is a recognized concern [12], and broader GI dysfunction is common after SCI [13]. Our results suggest that VSG may be particularly effective for SCI-associated obesity by simultaneously lowering body weight and reducing sweet-motivated behavior. The NTS activation profile provides a plausible neural marker for this effect and a target for future mechanistic or neuromodulatory interventions. Despite their translational relevance, these results should be interpreted with caution when extending to human SCI, as dietary decision-making in humans occurs within a substantially more complex sensory and psychosocial food environment than the controlled taste exposure used in this model. Moreover, species-specific differences in post-SCI metabolic remodeling, particularly with respect to skeletal muscle loss and energy expenditure, may influence the magnitude and nature of surgery and diet-related outcomes.

### 4.6. Limitations

The study has several limitations that may temper interpretation. (i) Only male Wistar rats were used in this initial study to reduce biological heterogeneity and allow mechanistic resolution of the combined effects of spinal cord injury (SCI) and vertical sleeve gastrectomy (VSG). Both SCI outcomes and bariatric surgery-induced metabolic adaptations are strongly influenced by sex hormones, and inclusion of both sexes in a high-dimensional design would substantially increase variance and reduce statistical power to detect primary effects and interactions. Restricting the cohort to males enabled isolation of a coherent physiological response profile prior to sex-specific extension in future studies. (ii) Behavioral assays were limited to brief-access licking and 24 h two-bottle choice of only one, and somewhat low, concentration of sucrose solution. Given this limitation and the longitudinal study design, analysis of more variable salt licking data and two-bottle choice testing following abdominal surgery were underpowered for meaningful conclusions. Future studies using progressive ratio or conditioned flavor preference paradigms would clarify contributions of motivation versus palatability. (iii) cFos is time-averaged and cell-type agnostic; therefore, future work should co-label GLP-1, catecholaminergic, and CGRP populations and map projections to parabrachial and hypothalamic targets. (iv) We did not quantify gut hormones, gastric emptying, bile acids, or microbiota, candidate mediators of the VSG phenotype [21]. (v) Although motor ability can influence licking, group differences localized to motivational microstructure and lick frequency were largely preserved; nevertheless, complementary measures of oromotor functions would be useful.

### 4.7. Future Directions

Mechanistic studies should (1) identify DVC neuronal populations engaged by sucrose after SCI alone and in combination with VSG (e.g., Fos co-labeling); (2) test causal roles of vagal afferents, as well as efferents, in mediating post-VSG sucrose suppression; (3) profile gut hormones, bile acids, and microbiota longitudinally across SCI, HFD, and VSG phases [21]; and (4) extend analyses to parabrachial and mesolimbic targets to map how SCI affects hindbrain signals to recalibrate reward circuits. Translationally, pilot studies in individuals with SCI could quantify taste intensity/pleasantness, food choice, and gut–brain peptides before and after VSG or GLP-1RA therapy, with autonomic and GI motility phenotyping to define risk–benefit boundaries [12,13]. Once the underlying mechanisms are revealed, non-surgical alternatives to bariatric surgery could be explored with similar benefits to the SCI population.

## 5. Conclusions

Here, we show that the combination of SCI and an obesogenic HFD led to greater impairments in sweet taste response compared to non-SCI animals. Importantly, VSG was more effective in restoring taste function in SCI rats than in non-SCI rats. Specifically, SCI rats exhibited reduced licking responses to sucrose at higher concentrations and heightened responsiveness to low concentrations of sodium chloride despite showing no differences in long-term preference for sucrose. Critically, VSG reduced sucrose response in both SCI and non-SCI rats, but the effect was significantly more pronounced in the SCI cohort. These observations together with augmented sucrose-evoked DVC activation, support a model in which post-surgical improvements in early hindbrain sensory–visceral signals constrain intake and normalize sucrose response.

The findings presented here provide the first evidence that SCI not only predisposes individuals to diet-induced obesity through alterations in taste function but also that VSG may more effectively ameliorate such deficits, particularly in normalizing sweet taste responses, than in non-SCI counterparts with obesity. This study highlights the advantages of using an animal model of SCI to examine primary taste functions and preferences without the confounding psychosocial factors (e.g., stress, lifestyle changes, inactivity, social isolation, etc.) present in human populations. This study supports the conceptual model that SCI modifies taste responsivity contributing to risk of developing obesity, which can be prevented or reversed by weight loss surgery. Nonetheless, future research is warranted to evaluate the translational relevance of these results in human subjects and to further elucidate the underlying neural and hormonal mechanisms underlying these effects.

## Data Availability

Data is publicly available under the Creative Commons License at https://doi.org/10.6084/m9.figshare.30836837.

## Abbreviations

The following abbreviations are used in this manuscript:

SCI	Spinal cord injury
VSG	Vertical sleeve gastrectomy
GI	Gastrointestinal
HFD	High-fat diet
DVC	Dorsal vagal complex
2BC	Two-bottle choice test
NTS	Nucleus of the solitary tract
CGRP	Calcitonin Gene-Related Peptide
GLP1	Glucagon-like Peptide 1
GLP1-RA	Glucagon-like Peptide 1 Receptor Agonist
BMI	Body mass index
